# Supplementary material for: Epydemix: An open-source Python package for epidemic modeling with integrated approximate Bayesian calibration
Source: PLoS Comput Biol. 2025 Nov 19;21(11):e1013735. doi: 10.1371/journal.pcbi.1013735 (PMC12654906; doi:10.1371/journal.pcbi.1013735)
Supplement: S1 Text — (PDF) [file pcbi.1013735.s001.pdf]

Supplementary Information for *Epydemix: An open-source Python package for epidemic modeling with integrated approximate Bayesian calibration*

Nicolò Gozzi[1,2,*], Matteo Chinazzi[2,3], Jessica T. Davis[2], Corrado Gioannini[1], Luca Rossi[1], Marco Ajelli[4], Nicola Perra[5,2,6], Alessandro Vespignani[2,1]

[1] ISI Foundation, Turin, Italy

[2] Laboratory for the Modeling of Biological and Socio-technical Systems, Northeastern University, Boston, Massachusetts, United States of America

[3] The Roux Institute, Northeastern University, Portland, Maine, United States of America

[4] Laboratory for Computational Epidemiology and Public Health, Department of Epidemiology and Biostatistics, Indiana University School of Public Health, Bloomington, Indiana, United States of America

[5] School of Mathematical Sciences, Queen Mary University of London, United Kingdom

[6] The Alan Turing Institute, London, United Kingdom

[*] nicolo.gozzi@isi.it

Contents

S1 Additional information on the implementation **2**

S1.1 Stochastic SIR Model: Example 2

S1.2 Multinomial transitions 4

S2 ABC Algorithms **6**

S3 Modeling COVID-19 in Massachusetts **8**

S3.1 Epidemic model 8

S3.2 Model calibration 10

S1 Additional information on the implementation

S1.1 Stochastic SIR Model: Example

As example for the Epydemix simulation paradigm, we consider the prototypical SIR model. Susceptible and healthy individuals are placed in the S compartment. Through interactions with infected individuals (I), they can become infected with probability β. After the infectious period (μ^{-1}), infected individuals transition to the recovered compartment (R), where they can no longer spread the disease or become reinfected. This model can be translated into the following stochastic processes, which can be iterated computationally:

$$\begin{aligned} S_k(t+\delta t) &= S_k(t) - Bin(S_k(t), \lambda_r(k,t)), \\ I_k(t+\delta t) &= I_k(t) - Bin(I_k(t), \mu_r), \\ R_k(t+\delta t) &= R_k(t) + Bin(I_k(t), \mu_r), \end{aligned} \tag{1}$$

where $\lambda_r(k,t)$ and μ_r are the transition probabilities obtained by transforming rates into risks. Specifically, $\mu_r = 1 - e^{-\mu\delta t}$ and $\lambda_r(k,t) = 1 - e^{-\lambda(k,t)\delta t}$, where $\lambda(k,t)$ represents the force of infection for group k, i.e., the per-capita rate at which susceptible individuals in group k transition to the infected compartment. In Epydemix, forces of infection (i.e., transition rates of mediated transitions) are computed by accounting for interactions with individuals in mediating compartment through a contact matrix $\mathbf{C}$. The element $C_{kk'}$ of the matrix describes the average number of contacts an individual in group k has with individuals in group k' per unit of time (set here to 1 day). In this context, $\lambda(k,t)$ is calculated as:

$$\lambda(k,t) = \beta \sum_{k'=1}^{K} C_{kk'} \frac{I_{k'}(t)}{N_{k'}}, \tag{2}$$

where $N_{k'}$ is the total number of individuals in group k'. In Epydemix, the simulation of this process is achieved by iteratively solving the equations in System 1, calculating the appropriate transition probabilities for binomial sampling at each step. Algorithm S1 provides the pseudocode for the simulation of this system.

Algorithm S1 Stochastic SIR Model Simulation

Input:
  $S_k(0)$, $I_k(0)$, $R_k(0)$: Initial compartment values for all groups k
  $C_{kk'}$: Contact matrix
  β: Transmission rate
  μ: Recovery rate
  δt: Time step
  T: Total simulation time
Output: $S_k(t)$, $I_k(t)$, $R_k(t)$ for all t
Initialize $S_k(0)$, $I_k(0)$, $R_k(0)$ for all groups k
Set $t \leftarrow 0$
while $t < T$ **do**
  for each group k **do**
    Compute force of infection: $\lambda(k,t) \leftarrow \beta \sum_{k'=1}^{K} C_{kk'} \frac{I_{k'}(t)}{N_{k'}}$
    Compute infection probability: $\lambda_r(k,t) \leftarrow 1 - e^{-\lambda(k,t)\delta t}$
    Sample new infections: new_infections $\leftarrow$ $\mathrm{Bin}(S_k(t), \lambda_r(k,t))$
    Compute recovery probability: $\mu_r \leftarrow 1 - e^{-\mu \delta t}$
    Sample recoveries: new_recoveries $\leftarrow$ $\mathrm{Bin}(I_k(t), \mu_r)$
    Update compartments:

$$
\begin{aligned}
S_k(t+\delta t) &\leftarrow S_k(t) - \text{new\_infections} \\
I_k(t+\delta t) &\leftarrow I_k(t) + \text{new\_infections} - \text{new\_recoveries} \\
R_k(t+\delta t) &\leftarrow R_k(t) + \text{new\_recoveries}
\end{aligned}
$$

  end for
  Increment time: $t \leftarrow t + \delta t$
end while
return $S_k(t)$, $I_k(t)$, and $R_k(t)$ for all t

S1.2 Multinomial transitions

As explained in the main text, simulations in Epydemix are stochastic and follow a chain binomial approach to model transitions between compartments. That means that, at each time step, the number of individuals transitioning from compartment X_k to Y_k is drawn from a binomial distribution, $\text{Bin}(X_k, p_{X_k \to Y_k})$, where X_k denotes the number of individuals currently in the source compartment, and $p_{X_k \to Y_k}$ represents the probability of transition within the interval δt. By default, transition probabilities are computed by converting transition rates (i.e., $r_{X_k \to Y_k}$) into discrete-time risks using the standard exponential transformation:

$$p_{X_k \to Y_k} = 1 - e^{-r_{X_k \to Y_k} \delta t}$$

When a compartment has multiple possible destination states, transitions are modeled using a multinomial extension of the chain binomial process.

The total rate of exiting X_k is given by: $r^{\text{tot}}_{X_k} = \sum_{i=1}^{m} r_{X_k \to Y_k^i}$. The corresponding probability of leaving X_k during the time interval δt is then $p^{\text{exit}}_{X_k} = 1 - e^{-r^{\text{tot}}_{X_k} \delta t}$. Each destination-specific transition probability is computed as the fraction of the total exit probability proportional to its rate:

$$p_{X_k \to Y_k^i} = p^{\text{exit}}_{X_k} \frac{r_{X_k \to Y_k^i}}{r^{\text{tot}}_{X_k}}, \quad i = 1, \ldots, m$$

Finally, the vector of transitions is drawn from a multinomial distribution:

$$(N_{X_k \to Y_k^1}, \ldots, N_{X_k \to Y_k^m}, N_{X_k \to X_k}) \sim \text{Mult}\left(X_k, p_{X_k \to Y_k^1}, \ldots, p_{X_k \to Y_k^m}, 1 - p^{\text{exit}}_{X_k}\right),$$

where the last term corresponds to individuals who remain in X_k during δt.

Example: Infection with Two Possible Outcomes. Consider an extension of the SIR model in which susceptible individuals (S_k) can become infected in two distinct ways upon exposure: they may develop either an asymptomatic infection ($I_{a,k}$) or a symptomatic infection ($I_{s,k}$). The two infection types differ in their transmissibility, characterized by infection rates β_a and β_s, respectively, with $\beta_s > \beta_a$.

The total force of infection for group k is the sum of the contributions from both infectious compartments:

$$\lambda(k,t) = \beta_a \sum_{k'=1}^{K} C_{kk'} \frac{I_{a,k'}(t)}{N_{k'}} + \beta_s \sum_{k'=1}^{K} C_{kk'} \frac{I_{s,k'}(t)}{N_{k'}}. \quad (3)$$

When a susceptible individual becomes infected during δt, they are assigned to either the asymptomatic or symptomatic compartment according to the probabilities π_a and $\pi_s = 1 - \pi_a$, which represent the fractions of infections resulting in each outcome.

At each time step, the multinomial process governing transitions out of S_k is:

$$(N_{S_k \to I_{a,k}},\, N_{S_k \to I_{s,k}},\, N_{S_k \to S_k}) \sim \mathrm{Mult}\big(S_k,\, p_{S_k \to I_{a,k}},\, p_{S_k \to I_{s,k}},\, 1 - p_{S_k}^{\mathrm{exit}}\big)\,, \tag{4}$$

where the total exit probability from S_k is

$$p_{S_k}^{\mathrm{exit}} = 1 - e^{-\lambda(k,t)\delta t}, \tag{5}$$

and the destination-specific probabilities are given by:

$$p_{S_k \to I_{a,k}} = \pi_a \, p_{S_k}^{\mathrm{exit}}, \qquad p_{S_k \to I_{s,k}} = \pi_s \, p_{S_k}^{\mathrm{exit}}. \tag{6}$$

Each infected compartment then evolves independently according to its own recovery rate μ_a or μ_s:

$$\begin{aligned} I_{a,k}(t+\delta t) &= I_{a,k}(t) - \mathrm{Bin}(I_{a,k}(t),\, \mu_{a,r}), \\ I_{s,k}(t+\delta t) &= I_{s,k}(t) - \mathrm{Bin}(I_{s,k}(t),\, \mu_{s,r}), \end{aligned} \tag{7}$$

where $\mu_{a,r} = 1 - e^{-\mu_a \delta t}$ and $\mu_{s,r} = 1 - e^{-\mu_s \delta t}$.

This setup illustrates how the multinomial approach allows one to flexibly handle multiple infection pathways from a single source compartment, each with distinct transmission characteristics.

S2 ABC Algorithms

In this section we provide pseudocodes for the three ABC algorithms: ABC Rejection Algorithm (Algorithm S2), ABC Algorithm with Simulation Budget (Algorithm S3), ABC-SMC Algorithm (Algorithm S4).

Algorithm S2 ABC Rejection Algorithm

Input:
  P: Population size (number of accepted parameter sets)
  $\pi(\boldsymbol{\theta})$: Prior distribution of parameters
  $d(\cdot,\cdot)$: Distance metric
  ϵ: Tolerance threshold
  $f(\cdot|\boldsymbol{\theta})$: Simulator model
  $\mathbf{y}_{\text{obs}}$: Observed data
Output: Set of accepted parameters $\{\boldsymbol{\theta}_i\}_{i=1}^{P}$
Initialize an empty set of accepted parameters: Accepted $\leftarrow \{\}$
while $|\text{Accepted}| < P$ **do**
    Sample a parameter set $\boldsymbol{\theta}_i$ from the prior distribution $\pi(\boldsymbol{\theta})$
    Simulate $\mathbf{y}_i \sim f(\cdot|\boldsymbol{\theta}_i)$
    Compute the distance $d(\mathbf{y}_i, \mathbf{y}_{\text{obs}})$
    if $d(\mathbf{y}_i, \mathbf{y}_{\text{obs}}) < \epsilon$ **then**
        Add $\boldsymbol{\theta}_i$ to Accepted
    end if
end while
return Accepted

Algorithm S3 ABC Algorithm with Simulation Budget

Input:
  B: Total simulation budget (number of total simulations allowed)
  X: Percentage of best trajectories to select
  $\pi(\boldsymbol{\theta})$: Prior distribution of parameters
  $d(\cdot,\cdot)$: Distance metric
  $f(\cdot|\boldsymbol{\theta})$: Simulator model
  $\mathbf{y}_{\text{obs}}$: Observed data
Output: Set of accepted parameters $\{\boldsymbol{\theta}_i\}_{i=1}^{\lfloor B\cdot X/100\rfloor}$
Initialize an empty set of parameter-distance pairs: Candidates $\leftarrow \{\}$
for $i = 1$ to B **do**
    Sample a parameter set $\boldsymbol{\theta}_i$ from the prior distribution $\pi(\boldsymbol{\theta})$
    Simulate $\mathbf{y}_i \sim f(\cdot|\boldsymbol{\theta}_i)$
    Compute the distance $d(\mathbf{y}_i, \mathbf{y}_{\text{obs}})$
    Add pair $(\boldsymbol{\theta}_i, d(\mathbf{y}_i, \mathbf{y}_{\text{obs}}))$ to Candidates
end for
Sort Candidates by the distance $d(\mathbf{y}_i, \mathbf{y}_{\text{obs}})$ in ascending order
Select the top $\lfloor B \cdot X/100\rfloor$ parameter sets based on the sorted distances
return Selected parameter sets $\{\boldsymbol{\theta}_i\}_{i=1}^{\lfloor B\cdot X/100\rfloor}$

Algorithm S4 ABC-SMC Algorithm

Input:
  P: Number of particles
  δ: Sequence of tolerance levels $\delta_1 > \delta_2 > \cdots > \delta_T$
  $\pi(\boldsymbol{\theta})$: Prior distribution
  $f(\cdot|\boldsymbol{\theta})$: Simulator model
  $d(\cdot,\cdot)$: Distance function
  $K(\cdot|\cdot)$: Perturbation kernel
  $\mathbf{y}_{\text{obs}}$: Observed data
Output: Posterior distribution approximations $\{\boldsymbol{\theta}_i^{(t)}\}_{i=1}^P$ for each $t = 1, \ldots, T$
for $t = 1$ to T **do**
  if $t = 1$ **then**
    for $i = 1$ to P **do**
      repeat
        Sample $\boldsymbol{\theta}_i^{(1)} \sim \pi(\boldsymbol{\theta})$
        Simulate $\mathbf{y}_i \sim f(\cdot|\boldsymbol{\theta}_i^{(1)})$
      until $d(\mathbf{y}_i, \mathbf{y}_{\text{obs}}) \leq \epsilon_1$
      Set weight $w_i^{(1)} = \frac{1}{P}$
    end for
  else
    for $i = 1$ to P **do**
      repeat
        Sample $\boldsymbol{\theta}_i^{(t-1)} \sim \{\boldsymbol{\theta}_j^{(t-1)}\}_{j=1}^N$ with weights $w_j^{(t-1)}$
        Perturb $\boldsymbol{\theta}_i^{(t)} \sim K(\boldsymbol{\theta}|\boldsymbol{\theta}_i^{(t-1)})$
        Simulate $\mathbf{y}_i \sim f(\cdot|\boldsymbol{\theta}_i^{(t)})$
      until $d(\mathbf{y}_i, \mathbf{y}_{\text{obs}}) \leq \epsilon_t$
      Compute weight $w_i^{(t)} = \frac{\pi(\boldsymbol{\theta}_i^{(t)})}{\sum_{j=1}^N w_j^{(t-1)} K(\boldsymbol{\theta}_i^{(t)}|\boldsymbol{\theta}_j^{(t-1)})}$
    end for
  end if
  Normalize weights: $w_i^{(t)} \leftarrow \frac{w_i^{(t)}}{\sum_{j=1}^N w_j^{(t)}}$ for $i = 1, \ldots, P$
end for

S3 Modeling COVID-19 in Massachusetts

S3.1 Epidemic model

In this section, we provide a detailed description of the epidemic model introduced in the main text, which is used to simulate the spread of COVID-19 in Massachusetts, US, during early 2020.

The natural history of the disease is represented using an SEIR-like compartmental model, augmented with additional compartments to account for disease-related deaths. We stratify the population into 10 age groups k (i.e., $[0-9, 10-19, 20-24, 25-29, 30-39, 40-49, 50-59, 60-69, 70-79, 80+]$), where each group is characterized by a population size N_k, reflecting the real demographic distribution. Additionally, we incorporate the contact matrix $\mathbf{C} \in \mathbb{R}^{K \times K}$, where the element $C_{i,j}$ represents the average number of daily effective contacts between individuals in age group i and those in age group j [1].

The transition rate of susceptible individuals S to the exposed state E, known as the force of infection, is given by:

$$\lambda(k,t) = \beta s(t) r(t) \sum_{k'=1}^{K} C_{kk'} \frac{I_{k'}(t)}{N_{k'}}. \tag{8}$$

Here, the infection rate is assumed to be proportional to the fraction of infectious individuals in each age group, where β denotes transmissibility of the disease. The expression also includes a seasonality modulation term $s(t)$, which accounts for variations in humidity, temperature, and other environmental factors that influence both transmissibility and contact patterns [2, 3]. The seasonality function is defined as:

$$s_i(t) = \frac{1}{2} \left[\left(1 - \frac{s_{min}}{s_{max}}\right) \sin\left(\frac{2\pi}{365}(t - t_{max,i}) + \frac{\pi}{2}\right) + 1 + \frac{s_{min}}{s_{max}} \right] \tag{9}$$

where i represents the Hemisphere, and $t_{max,i}$ corresponds to the time of maximum seasonality. We set this to January 15th in the Northern Hemisphere and shift it by six months in the Southern Hemisphere, assuming no seasonality in tropical regions. The maximum seasonality factor is fixed at $s_{max} = 1$, while s_{min} is treated as a free parameter [3, 4].

The force of infection is further modulated by the contact variation factor $r(t)$, which is derived from the Community Mobility Report published by Google LLC [5]. This dataset provides percentage changes in mobility and visits to various locations over time. Since our model does not distinguish between different locations (i.e., contexts), we introduce a single mobility parameter, $m(t)$, representing the average percentage reduction in visits across all locations (excluding parks due to their atypical behavior). The mobility factor is converted into an effective contact reduction parameter as:

$$r(t) = \left(1 - \frac{|m(t)|}{100}\right)^2. \tag{10}$$

The rationale behind this formulation is that under a homogeneous mixing assumption, the number of

potential contacts C scales quadratically with population size, i.e., $C = N(N-1)/2 \sim N^2$. If the number of individuals present in a location is reduced to $N'(t) = (1 - m(t)/100)N$, the number of potential contacts follows $C'(t) \sim N'(t)^2 = (1 - m(t)/100)^2 N^2$, yielding the contact reduction factor $r(t) = C'(t)/C$.

Exposed individuals progress to the infectious state I at rate ϵ, which is the inverse of the latent period. Infectious individuals then transition to the removed state R at rate μ, the inverse of the infectious period. The removed compartment includes individuals who are no longer infectious.

To model COVID-19 mortality, we track the transitions from I_k to R_k. A fraction of these transitions, determined by age-stratified estimates of the Infection Fatality Rate (IFR) from Ref. [6], leads to the $D_{0,k}$ compartment, which represents deceased individuals. Since death does not occur immediately after recovery due to hospitalization, isolation, and reporting delays, we introduce additional compartments $(D_{1,k}, D_{2,k}, D_{3,k}, D_{4,k})$. Individuals transition between these compartments at a rate of $4/\Delta$, where Δ is the average delay (in days) between recovery and recorded death. The number of individuals transitioning from $D_{3,k}$ to $D_{4,k}$ on day t corresponds to the reported deaths on that day. The introduction of multiple $D_{i,k}$ compartments ensures that the delay distribution follows an Erlang distribution with shape parameter $n = 4$ [7].

The disease progression is simulated using stochastic chain binomial processes, as described above. The model can be expressed by the following stochastic process that can be conveniently iterated computationally:

$$
\begin{aligned}
S_k(t+\delta t) &= S_k(t) - Bin(S_k(t), \lambda_r(k,t)), && (11)\\
E_k(t+\delta t) &= E_k(t) + Bin(S_k(t), \lambda_r(k,t)) - Bin(E_k(t), \epsilon_r), && (12)\\
I_k(t+\delta t) &= I_k(t) + Bin(E_k(t), \epsilon_r) - Mult_1(I_k(t), 1 - e^{-\mu(1-IFR_k)\delta t}, 1 - e^{-\mu IFR_k \delta t}) \\
&\quad - Mult_2(I_k(t), 1 - e^{-\mu(1-IFR_k)\delta t}, 1 - e^{-\mu IFR_k \delta t}), \\
R_k(t+\delta t) &= R_k(t) + Mult_1(I_k(t), 1 - e^{-\mu(1-IFR_k)\delta t}, 1 - e^{-\mu IFR_k \delta t}), \\
D_{0,k}(t+\delta t) &= D_{0,k}(t) + Mult_2(I_k(t), 1 - e^{-\mu(1-IFR_k)\delta t}, 1 - e^{-\mu IFR_k \delta t}) - Bin(D_{0,k}(t), 4/\Delta), \\
D_{1,k}(t+\delta t) &= D_{1,k}(t) + Bin(D_{0,k}(t), 4/\Delta) - Bin(D_{1,k}(t), 4/\Delta), \\
D_{2,k}(t+\delta t) &= D_{2,k}(t) + Bin(D_{1,k}(t), 4/\Delta) - Bin(D_{2,k}(t), 4/\Delta), \\
D_{3,k}(t+\delta t) &= D_{3,k}(t) + Bin(D_{2,k}(t), 4/\Delta) - Bin(D_{3,k}(t), 4/\Delta), \\
D_{4,k}(t+\delta t) &= D_{4,k}(t) + Bin(D_{3,k}(t), 4/\Delta)
\end{aligned}
$$

For the above contagion process, the basic reproduction number is $R_0 = \rho(\tilde{\mathbf{C}})\beta/\mu$, where $\tilde{C_{ij}} = C_{ij} N_i / N_j$, $\rho(\cdot)$ is the spectral radius [8], and the generation time of the disease is $T_G = \epsilon^{-1} + \mu^{-1}$.

S3.2 Model calibration

The model is calibrated to weekly reported deaths from Ref. [9] using the ABC-SMC algorithm, employing 10 generations with $1,000$ accepted particles per generation. The free parameters and their prior distributions, all assumed to follow uniform distributions, are: the basic reproduction number $R_0 \sim U(1.5, 6.0)$, the seasonality parameter $s(t) \sim U(0.6, 1.0)$, the delay between recovery and death $\Delta \sim U(7, 35)$, and the initial number of infected individuals across age groups $I_0 \sim U(10, 10000)$.

Figure S1 presents the estimated posterior distributions for these parameters.

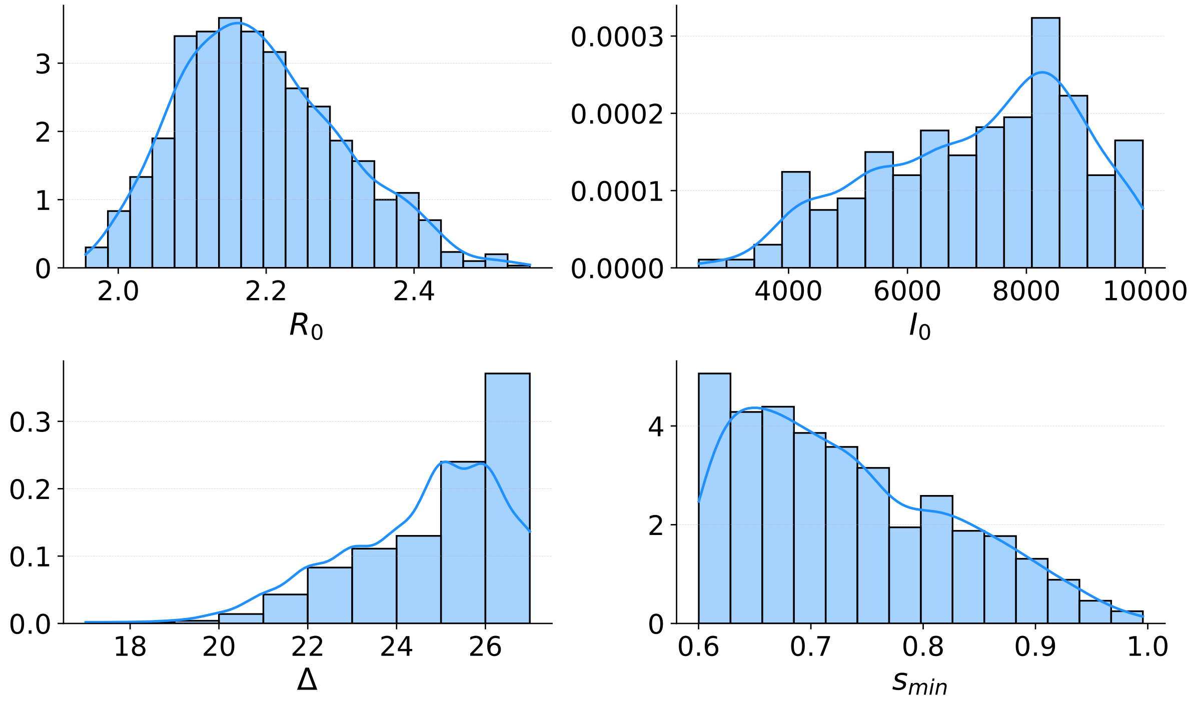

Figure S1: Posterior distributions of free epidemiological parameters inferred via ABC-SMC calibration. The histograms represent the sampled posterior distributions for the basic reproduction number (R_0), initial number of infected individuals (I_0), average delay between recovery and death (Δ), and minimum seasonality factor (s_{min}). The blue curves indicate kernel density estimates (KDE) of the posterior distributions.

S4 Related work

We provide a description of the existing tools mentioned in the main text. See Table S1 for a schematic summary. To help streamlining the narrative, we group them based on the analytical framework(s) adopted. The first class are tools based on compartmental models. COVID-19 Scenarios provides an easy to use web application that allows setting a range of parameters, interventions, and inspect the outcomes [10]. The framework allows setting the scale of the population under exam extracting features from a given set of countries. The tool is thought for COVID-19 and does not offer support for the calibration phase. SimCOVID is built via Simulink, and provides calibration capabilities via MATLAB [11]. The framework has been developed for COVID-19, but it comes equipped with several compartmental models hence can be used to simulate the spreading of similar diseases.

GLEAMviz is a global metapopulation model accessible via a GUI [12]. It accounts for long-range (i.e., air transportation) and short-range (i.e., commuting) mobility patterns. GLEAMviz takes the compartmental structure of the disease under investigation as input, together with a range of other parameters such as the geographical location of the epicenter of the epidemic. GLEAMviz allows simulating and visualizing the global spreading of infectious diseases but it does not provide direct support for calibration.

Among the agent-based models we find a wide variety of approaches. COVASIM is written in Python and developed to model the spreading of COVID-19 as well as to test the impact of different interventions [13]. Interestingly, it provides support for modeling intra-host viral dynamics. COVASIM comes with the data necessary to run the model in a few geographical locations. Though the framework has been developed targeting the COVID-19 Pandemic, the code is open-source and it can be adapted to similar diseases. While COVASIM provides loss functions, the fitting to particular epidemic data is left to the user via the integration with other tools. Agents.jl is a framework written in Julia that simulates dynamical processes (such as disease spreading) taking place among agents interacting in different settings such as grids, graphs, and particular geographical locations initialized from OpenStreetMap [14]. Agents.jl supports parameters exploration and calibration. FluTE is an agent-based simulation model for influenza epidemics [15]. It is written in C++ and has been developed to reproduce the spreading of influenza in the USA. In terms of geographical scale, FluTE can go from single small communities to the whole USA. Calibration is not directly supported, though some key parameters of the model have been fitted to reproduce a few historical seasons. FRED is a framework written in C++ to simulate the spreading of respiratory diseases and evaluate the impact of both pharmaceutical and non-pharmaceutical interventions (NPIs) [16]. It comes with synthetic data needed to run it in any US state and county as well as some regions in India. The tool does not provide support for calibration. COMOKIT has been developed to model the spreading of SARS-CoV-2 and evaluate the impact of mitigation policies at the scale of a city [17]. It is written with the GAMA platform and does not provide explicit calibration

functionalities. BESSIE is a modeling framework written in Python designed to simulate the spreading of SARS-CoV-2-like epidemics [18]. Interestingly, it has been developed to study behavioral adaption of target populations as well as the impact of NPIs. BESSIE allows modeling specific behaviors of single agents and assess their impact on the disease spreading. The geographical scale of analysis is set by the input data. BESSIE does not provide calibration functions. Pyfectious is written in Python and designed to model the spreading of respiratory diseases at the city level [19]. Interestingly, it features a generative model able to create realistic structured synthetic populations requiring less inputs with respect to similar frameworks. Furthermore, it features reinforcement learning algorithms to infer optimal intervention policies given a specified target function such as reducing severity. Pyfectious provides support to the estimation of parameters linked to the discovery of such optimal optimization strategies. OpenABM-Covid19 is written in C with Python and R interfaces [20]. The geographical scale of analysis is that of a city. By default the model builds synthetic populations with the characteristics of the UK, but it can be extended to other locations. The tool has been developed to model the spreading of SARS-CoV-2 and study the impact of intervention strategies such as contact tracing. OpenABM-Covid19 does not provide direct support for the calibration phase. Sampy is a package written in Python to simulate the spreading of infectious diseases among agents embedded in space [21]. The framework allows considering different topologies describing the connections between the geographical units. It does not provide direct support for calibration.

Among existing network-based tools we find a wide variety of approaches. CSonNet is designed to simulate the contagion processes on contact networks [22]. It targets mid-sized graphs up to 10^5 nodes. CSonNet is written in Python and does not provide support for calibration. VTES is an educational tool written in Python [23]. Agents move and interact in a 2D plane. VTES has not the ambition of a realistic epidemic modeling framework but it is thought as educational tool to showcase the impact of contacts patterns on the spreading of diseases. GEMFsim is a tool that allows modeling the spreading of contagion processes on a range of network types (including multilayer networks) [24]. Interestingly, it can be used in MATLAB, R, Python, and C. GEMFsim does not provide support for calibration. SimpactCyan has been developed to model the spreading of HIV unfolding on dynamic networks [25]. It is written in C++, but usable via R and Python interfaces. SimpactCyan allows to study the impact of treatment and prevention policies. The tool does not provide calibration functions. Epinet simulates the spreading of diseases unfolding on exponential random graphs [26]. It is written in R and allows estimating the parameters of a contact network from epidemic data. EoN provides a range of functionalities to simulate contagion processes on networks [27]. The tool supports a range of implementations and functions to visualize the simulated spreading process on networks. However, it does not offer direct support for calibration. Ref. [28] provides a fast implementation of SIR models unfolding on temporal networks. The code is available in both C and Python. No support is provided for calibration.

Finally, we find tools that allow the implementation of a range of model types. EpiFire offers the possibility to model both simple compartmental and network-based models. The framework is written in C++, but comes along with a GUI. EpiFire does not support the calibration phase, but provides a range of methods for the analytical evaluation of relevant metrics such as R_0. MEmilio provides support for the implementation of a variety of models type such as compartmental, agent-based, and metapopulation [29]. The core of the tool is written C++. Python is used for plotting and data manipulation. Furthermore, some C++ routines can be also called directly from Python. The geographical scale of analysis can be changed according to the study. Though the tool is fairly general, the published version provides a range of input data only for Germany. Parameters' calibration is not supported directly, but the framework can easily accommodate integrations with external tools. Eir is a Python framework to simulate the spreading of infectious diseases on both compartmental and different types of spatial models [30]. It does not offer direct support for calibration. Epilearn is a Python framework that allows for the implementation of statistical (e.g., auto-regressive), machine learning (e.g., XGBoost, LSTM, DLinear), compartmental, and spatial models. It is designed to tackle two different tasks: forecasting and epidemic source detection. As such, it provides direct support for calibration. EpiModel is an R package to model the spreading of infectious diseases using compartmental, agent-based, and network models [31]. Interestingly, it allows considering temporal exponential random graphs which are fitted on empirical data. Finally, Ref. [32] provides a range of methods to implement compartmental and network-based models in Python. The goal is to help with the implementation of various models rather than their calibration to real data.

Table S1: Summary of epidemic-modeling software tools reviewed in the literature.

**Name**	**Type**	**Description**	**Disease**	**Technology**	**Support for Calibration**	**Reference**
Epydemix	Compartmental	Python package for stochastic epidemic simulation and calibration	General	Python	Yes (via Approximate Bayesian Computation)	/
COVID-19 Scenarios	Compartmental	Interactive web app for exploring COVID-19 epidemic scenarios	COVID-19	Web GUI	No (exploration only)	[10]
SimCOVID	Compartmental	Simulink/MATLAB model with calibration via optimization	COVID-19	MATLAB / Simulink	Yes (via MATLAB tools)	[11]
GLEAMviz	Metapopulation	GUI for global epidemic simulation with air travel and commuting flows	General	C++ / GUI	No (scenario-based)	[12]
Sampy	Agent-based / Spatial	Modular Python library for stochastic, spatially explicit ABMs of epidemics with host movement, disease dynamics, and interventions	General	Python	No (focus on modular simulation and efficiency)	[21]
COVASIM	Agent-based	COVID-19 simulator with interventions and intra-host features	COVID-19	Python	Partial (user-defined via API)	[13]
Agents.jl	Agent-based / Hybrid	General-purpose agent-based modeling framework (grid, graph, OSM)	General	Julia	Yes (parameter search supported)	[14]
FluTE	Agent-based	Influenza simulator using synthetic U.S. populations	Influenza	C++	No	[15]
FRED	Agent-based	US-scale synthetic-population epidemic simulator	Respiratory diseases	C++ / Python	No	[16]
COMOKIT	Agent-based	City-level COVID-19 simulator on GAMA platform	COVID-19	GAMA (Java) / GUI	No	[17]
OpenABM-Covid19	Agent-based	SARS-CoV-2 model with contact-tracing and interventions	COVID-19	C / Python / R	No	[20]
BESSIE	Agent-based	Behavior-centric framework for epidemic simulation	SARS-CoV-2-like	Python	No	[18]
Pyfectious	Agent-based	City-level simulator with RL policy optimization	Respiratory diseases	Python	Partial (for RL optimization)	[19]
CSonNet	Network-based / Agent-based	Contagion simulation on contact networks	General	Python	No	[22]
VTES	Network-based	Toy model for contagion dynamics in moving agents	General	Python	No	[23]
GEMFsim	Network-based	Multilayer epidemic model simulator across languages	General	C / R / MATLAB / Python	No	[24]
SimpactCyan	Network-based	Dynamic-network simulator for HIV treatment and prevention	HIV	C++ / R / Python	No	[25]
Epinet	Network-based	Epidemic simulation on ERGMs with inference capabilities	General	R	Yes (ERGM inference)	[26]
EoN	Network-based	Toolkit for epidemic processes on networks with visualization tools	General	Python	No	[27]
Fast Temporal SIR	Network-based	Efficient SIR simulation on temporal networks	General	C / Python	No	[28]
EpiFire	Hybrid (Compartmental / Network)	Simulation and analytic evaluation via GUI	General	C++ / GUI	No	[33]
MEmilio	Hybrid (Multi-type)	Framework for agent-based, compartmental, and metapopulation models	General	C++ / Python	No (external calibration possible)	[29]
Eir	Hybrid (Compartmental / Spatial)	Python framework supporting multiple spatial model types	General	Python	No	[30]
Epilearn	Hybrid / ML-based	Python library integrating statistical, ML, and compartmental models	General	Python	Yes (built-in for forecasting)	[34]
EpiModel	Hybrid (Compartmental / Network / ABM)	R package for epidemic modeling with temporal ERGMs	General	R	Yes (network parameter estimation)	[31]
Mathematics of Epidemics (Kiss et al.)	Framework / Educational	Python framework for teaching and implementing epidemic models	General	Python	No	[32]

References

[1] Dina Mistry, Maria Litvinova, Ana Pastore y Piontti, Matteo Chinazzi, Laura Fumanelli, Marcelo FC Gomes, Syed A Haque, Quan-Hui Liu, Kunpeng Mu, Xinyue Xiong, et al. Inferring high-resolution human mixing patterns for disease modeling. Nature communications, 12(1):323, 2021.

[2] Ben S Cooper, Richard J Pitman, W John Edmunds, and Nigel J Gay. Delaying the international spread of pandemic influenza. PLoS Med, 3(6):e212, 2006.

[3] Stephen M Kissler, Christine Tedijanto, Edward Goldstein, Yonatan H Grad, and Marc Lipsitch. Projecting the transmission dynamics of SARS-CoV-2 through the postpandemic period. Science, 368(6493):860–868, 2020.

[4] Balcan, Duygu and Gonçalves, Bruno and Hu, Hao and Ramasco, José J. and Colizza, Vittoria and Vespignani, Alessandro. Modeling the spatial spread of infectious diseases: The GLobal Epidemic and Mobility computational model. Journal of Computational Science, 1(3):132–145, aug 2010.

[5] Google LLC "Google COVID-19 Community Mobility Reports". `https://www.google.com/covid19/mobility/`, 2020. Accessed: 2021-08-01.

[6] Robert Verity, Lucy C Okell, Ilaria Dorigatti, Peter Winskill, Charles Whittaker, Natsuko Imai, Gina Cuomo-Dannenburg, Hayley Thompson, Patrick GT Walker, Han Fu, et al. Estimates of the severity of coronavirus disease 2019: a model-based analysis. The Lancet infectious diseases, 20(6):669–677, 2020.

[7] Olga Krylova and David J. D. Earn. Effects of the infectious period distribution on predicted transitions in childhood disease dynamics. Journal of The Royal Society Interface, 10(84):20130098, 2013.

[8] Odo Diekmann, JAP Heesterbeek, and Michael G Roberts. The construction of next-generation matrices for compartmental epidemic models. Journal of the royal society interface, 7(47):873–885, 2010.

[9] Coronavirus cases and deaths, USA. `https://usafacts.org/visualizations/coronavirus-covid-19-spread-map/state/`, 2024. Accessed: 2025-01-16.

[10] Nicholas B Noll, Ivan Aksamentov, Valentin Druelle, Abrie Badenhorst, Bruno Ronzani, Gavin Jefferies, Jan Albert, and Richard A Neher. COVID-19 Scenarios: an interactive tool to explore the spread and associated morbidity and mortality of SARS-CoV-2. MedRxiv, pages 2020–05, 2020.

[11] Ismael Abdulrahman. SimCOVID: Open-source simulation programs for the COVID-19 outbreak. SN Computer Science, 4(1):20, 2022.

[12] Wouter Van den Broeck, Corrado Gioannini, Bruno Gonçalves, Marco Quaggiotto, Vittoria Colizza, and Alessandro Vespignani. The GLEaMviz computational tool, a publicly available software to explore realistic epidemic spreading scenarios at the global scale. BMC infectious diseases, 11:1–14, 2011.

[13] Cliff C Kerr, Robyn M Stuart, Dina Mistry, Romesh G Abeysuriya, Katherine Rosenfeld, Gregory R Hart, Rafael C Núñez, Jamie A Cohen, Prashanth Selvaraj, Brittany Hagedorn, et al. Covasim: an agent-based model of COVID-19 dynamics and interventions. PLOS Computational Biology, 17(7):e1009149, 2021.

[14] George Datseris, Ali R. Vahdati, and Timothy C. DuBois. Agents.jl: a performant and feature-full agent-based modeling software of minimal code complexity. SIMULATION, 100(10):1019–1031, 2024.

[15] Dennis L Chao, M Elizabeth Halloran, Valerie J Obenchain, and Ira M Longini Jr. FluTE, a publicly available stochastic influenza epidemic simulation model. PLoS computational biology, 6(1):e1000656, 2010.

[16] John J Grefenstette, Shawn T Brown, Roni Rosenfeld, Jay DePasse, Nathan TB Stone, Phillip C Cooley, William D Wheaton, Alona Fyshe, David D Galloway, Anuroop Sriram, et al. FRED (A Framework for Reconstructing Epidemic Dynamics): an open-source software system for modeling infectious diseases and control strategies using census-based populations. BMC public health, 13:1–14, 2013.

[17] Benoit Gaudou, Nghi Quang Huynh, Damien Philippon, Arthur Brugière, Kevin Chapuis, Patrick Taillandier, Pierre Larmande, and Alexis Drogoul. Comokit: A modeling kit to understand, analyze, and compare the impacts of mitigation policies against the covid-19 epidemic at the scale of a city. Frontiers in public health, 8:563247, 2020.

[18] Henning S Mortveit, Stephen Adams, Faraz Dadgostari, Samarth Swarup, and Peter Beling. BESSIE: A Behavior and Epidemic Simulator for Use With Synthetic Populations. arXiv preprint arXiv:2203.11414, 2022.

[19] Arash Mehrjou, Ashkan Soleymani, Amin Abyaneh, Samir Bhatt, Bernhard Schölkopf, and Stefan Bauer. Pyfectious: An individual-level simulator to discover optimal containment policies for epidemic diseases. PLOS Computational Biology, 19(1):e1010799, 2023.

[20] Robert Hinch, William JM Probert, Anel Nurtay, Michelle Kendall, Chris Wymant, Matthew Hall, Katrina Lythgoe, Ana Bulas Cruz, Lele Zhao, Andrea Stewart, et al. OpenABM-Covid19—An agent-based model for non-pharmaceutical interventions against COVID-19 including contact tracing. PLoS computational biology, 17(7):e1009146, 2021.

[21] Francois Viard, Emily Acheson, Agathe Allibert, Caroline Sauve, and Patrick Leighton. SamPy: A New Python Library for Stochastic Spatial Agent-Based Modeling in Epidemiology of Infectious Diseases. 2022.

[22] Joshua D Priest, Aparna Kishore, Lucas Machi, Chris J Kuhlman, Dustin Machi, and SS Ravi. Csonnet: An agent-based modeling software system for discrete time simulation. In 2021 Winter Simulation Conference (WSC), pages 1–12. IEEE, 2021.

[23] Danielle L Kurtin, Daniel AJ Parsons, and Scott M Stagg. VTES: a stochastic Python-based tool to simulate viral transmission: Introducing a modular simulation to demonstrate how a virus may spread among a population. F1000Research, 9:1198, 2020.

[24] Faryad Darabi Sahneh, Aram Vajdi, Heman Shakeri, Futing Fan, and Caterina Scoglio. GEMFsim: A stochastic simulator for the generalized epidemic modeling framework. Journal of computational science, 22:36–44, 2017.

[25] Jori Liesenborgs, Diana M Hendrickx, Elise Kuylen, David Niyukuri, Niel Hens, and Wim Delva. SimpactCyan 1.0: An open-source simulator for individual-based models in HIV epidemiology with R and Python interfaces. Scientific reports, 9(1):19289, 2019.

[26] Chris Groendyke and David Welch. epinet: An R package to analyze epidemics spread across contact networks. Journal of Statistical Software, 83:1–22, 2018.

[27] Joel C Miller and Tony Ting. Eon (epidemics on networks): a fast, flexible python package for simulation, analytic approximation, and analysis of epidemics on networks. arXiv preprint arXiv:2001.02436, 2020.

[28] Petter Holme. Fast and principled simulations of the SIR model on temporal networks. Plos one, 16(2):e0246961, 2021.

[29] Martin Joachim Kühn, Patrick Lenz, Agatha Schmidt, Wadim Koslow, Julia Bicker, René Schmieding, Martin Siggel, Sebastian Binder, Annette Lutz, Sascha Korf, et al. Software: MEmilio v1. 1.0-A high performance Modular EpideMIcs simuLatIOn software. Technical report, Population Health Sciences, 2024.

[30] Mathew Jacob. Eir: A Python Package for Epidemic Simulation. Journal of Open Source Software, 6(62):3247, 2021.

[31] Samuel M Jenness, Steven M Goodreau, and Martina Morris. EpiModel: an R package for mathematical modeling of infectious disease over networks. Journal of statistical software, 84:1–47, 2018.

[32] István Z Kiss, Joel C Miller, Péter L Simon, et al. Mathematics of epidemics on networks. Cham: Springer, 598(2017):31, 2017.

[33] Thomas Hladish, Eugene Melamud, Luis Alberto Barrera, Alison Galvani, and Lauren Ancel Meyers. EpiFire: An open source C++ library and application for contact network epidemiology. BMC bioinformatics, 13:1–12, 2012.

[34] Zewen Liu, Yunxiao Li, Mingyang Wei, Guancheng Wan, Max SY Lau, and Wei Jin. Epilearn: A python library for machine learning in epidemic modeling. arXiv preprint arXiv:2406.06016, 2024.
