# Supplementary material for: Epydemix: An open-source Python package for epidemic modeling with integrated approximate Bayesian calibration
Source: PLoS Comput Biol. 2025 Nov 19;21(11):e1013735. doi: 10.1371/journal.pcbi.1013735 (PMC12654906; doi:10.1371/journal.pcbi.1013735)
Supplement: Attachment — Submitted filename: Response to Reviewers Epydemix.pdf [file pcbi.1013735.s002.pdf]

Point-by-point response

Below we provide a rebuttal to all points raised by the referees. We report their comments, verbatim, in red and our replies in blue.

Reviewer #1

The paper presents a python package for the development and analysis of compartmental epidemic models, integrated with Bayesian parameter estimation methods. The package includes a number of features to ease model development, including assisted age-structure definition, time-varying parameters and time-dependent events, integration with real world demographic data.

I started reading the paper with a negative feeling. Developing a compartmental model from scratch in Python is not difficult, and also applying a parameter estimation method to it does not require too much work. However, while reading the paper, I completely changed my mind. The proposed python package is not a basic API for model development, it is conceived in a way that simplifies the work for the user and, at the same time, forces him/her to give a well-structured design to the model, favoring reusability and modification of the developed models by others. Apart from this, the package includes a number of features I found very interesting, such as the integration of real world demographic data. This is really nice: you develop a model and you want to test it on populations from different countries? You can do this easily. The integration of the parameter estimation method is, of course, another very useful feature. Finally, another aspect I liked of the paper is the explicit reference to open-source development. Very often, research software is released with open-source licenses just to make it freely available to the community. In this case, the authors explicitly say that it is released with an open-source license to stimulate community-based development. But, more importantly, it is by looking at the github repository that one understands that the aim is to open to external contributors (although for the moment only the first author committed there). Indeed, several commits have been done during the development (not only a final one, just to distribute the package), as for many OS projects there is a documentation on read-the-docs, and an issue opened by an external user and answered by the maintainer. These are small things but that show the real open-source attitude of the project.

We thank the reviewer for their insightful and encouraging feedback. As correctly noted, the goal of Epydemix is to provide a comprehensive tool that supports users throughout the entire modeling pipeline, from model development to integration with external data and parameter estimation. We are pleased that our efforts to design a framework that promotes structured, reusable, easily extendable models and to document our approach transparently have been recognized and appreciated.

Apart from all these positive aspects of the paper, there are also a number of things that should be improved. I list them below, mixing major and minor issues:

1) Related work [MAJOR]. The description of related work is definitely insufficient. A lot of modelling approaches are listed and cited without a proper comparison. For example, many agent-based and network based approaches are cited, and I think these kinds of approaches are nowadays particularly significant. Your framework instead is for the development of standard compartmental models. You should motivate your development choice and the fact that you plan to extend in the direction of metapopulation models (as said in the final discussion). Moreover, the description of related work about parameter estimation/model calibration is mostly missing. It consists of a single paragraph ("As mentioned, ...") with one only citation. You should significantly improve this part. During covid-19 a lot of papers have been published in which parameter estimation methods are applied to sir models: you should review them and compare the Bayesian approaches you adopted with those in those papers.

We thank the reviewer for this valuable comment. We agree that the related work section should better contextualize Epydemix within existing modeling frameworks and methodologies. We have substantially revised and expanded this section to address these points (Lines 85-133). In particular:

- In the revised manuscript, we have expanded this section to include a clearer comparison between Epydemix and other modeling frameworks, including agent-based and network-based approaches. While these methods are highly expressive and suitable for capturing individual-level heterogeneity and detailed contact structures, Epydemix focuses on providing a flexible and modular environment for developing and calibrating structured compartmental epidemic models. We have added an explicit motivation for this design choice. Despite their simpler structure, single-population structured compartmental models remain fundamental tools for real-time epidemic analysis, scenario modeling, and short-term forecasting, particularly when detailed data necessary to parameterize large-scale simulations are unavailable. Our aim is to facilitate transparent model definition, reproducible inference, and practical applications while maintaining a structure that can be readily extended toward more complex metapopulation or network-coupled systems, as discussed in the Discussion section

- We have improved the section by clarifying our methodological choices regarding parameter estimation compared with possible alternatives adopted in other contexts. We agree that a wide range of calibration approaches have been developed and applied during recent health emergencies. We note, however, that the manuscript is not proposing a new inference method which would indeed entail an extensive comparison with previous solutions. Rather, the package adopts off-the-self approximate Bayesian methods widely adopted in the context of

infectious disease models calibration. As such, we believe that adding a literature review on the subject (even just considering SIR-like models) is beyond the scope of the paper. Instead, we focused on describing the Bayesian methods implemented in Epydemix, their role within the broader modeling framework, and a motivation of our choice.

2) Line 147, I think "X = [S,I,R]" should be replaced by "X \in \{S,I,R\}"

We thank the reviewer for this comment. We revised notation as suggested (Line 187).

3) Line 155. Your library works only with stochastic models. I understand that stochastic models can be preferable for several reasons, but several times ODE based models could be better (at least for performance reasons). So, forcing stochastic modelling could be seen as a limitation. You should discuss this choice and possibly consider including ODE based simulation to be included in a future version of the package.

We thank the reviewer for this constructive comment. We agree that deterministic (i.e., ODE-based) models can be advantageous in some contexts, particularly when computational efficiency is a priority. At the same time, deterministic models present limitations such as the inevitable emergence of "fractional" individuals, and more importantly, the inability to capture stochastic fluctuations, critical especially in small populations, near-threshold dynamics, and at the beginning of an outbreak. For these reasons, we prioritized the stochastic implementation, despite its higher computational cost. In the revised version of the manuscript we added a discussion clarifying our design choice. Furthermore, we mentioned deterministic implementations as a possible extension in future releases of the package (Lines 662-668).

4) Lines 162-165. You should be more precise in describing these rates, possibly by formalizing their definition. In the case of the spontaneous transition, for example, it seems that the parameter provided by the modeler is directly used as rate (as is) while typically the rate is computed by multiplying the parameter by the size of the compartment…

The interpretation is correct: in the current implementation, the rate parameter provided by the modeler is used directly for spontaneous transitions. To improve clarity, we have revised the text to provide a more precise and formalized description of how rates are defined and applied within the multinomial sampling process. We have also updated the code to make this behavior more transparent and included a concrete example illustrating the computation of rates for different (default) transition types, to help readers in interpreting these parameters.

5) Whole section 3 [MAJOR]: the whole description of the design and implementation is in abstract terms. Understanding would be made easier by the addition of

examples throughout the text. Please, revise the whole section, trying to make it easier to read for the non-deeply-expert reader.

In the initial submission, we chose to keep Section 3 at a higher level of abstraction, using the results section to illustrate the concepts through concrete examples. Nonetheless, we acknowledge that this choice may have made the section less accessible to some readers. We have therefore revised Section 3 to include more illustrative explanations and examples, and to strengthen the connections between the methodological descriptions, the results section, and the Supplementary Information.

6) Line 170. "simulation parameters" Which parameters? not clear

These are the parameters used to perform the simulations, or, in other words, the transition rates (i.e., μ and β of the SIR model). We have added a clarification to the text (Lines 213-218).

7) Section 3.3.3. Are all these interventions and changes time dependent? (if not, clarify) It would be very useful to include changes that are condition-based, namely that are triggered when a given condition on the simulation state is satisfied (e.g., lockdown when ratio of infected is >10%). This would be very useful, I think.

We thank the reviewer for this valuable suggestion. Both the add_interventions and override_parameter methods currently take as input the start and end dates of interventions or parameter overrides. In the revised manuscript, we have made this point clearer. We agree that introducing condition-based changes triggered by specific states of the simulation would be a very useful extension. While a more advanced mechanism is already possible through the definition of behavioral transitions (e.g., individuals reducing their contact rates as infections grow see this tutorial for example), we are carefully considering how to integrate the reviewer's proposed feature in future releases.

Reviewer #2

The Article introduces Epydemix, an open-source Python package for the development and calibration of stochastic compartmental epidemic models. Contrary to previous works, it also integrates Approximate Bayesian Computation (ABC) to infer model parameters on observed data. The authors present various usage examples displaying the flexibility of their framework.

The article is extremely well written and enables professionals and researchers to easily employ the software. The examples provided are clearly described and effectively showcase the potential of the framework. The theoretical assumptions underlying the epidemic modeling are explicitly stated and fully consistent with the objectives of the work. I believe this publication will greatly benefit the entire community.

We thank the reviewer for these very positive and encouraging remarks. We are pleased that the clarity, structure, and accessibility of the manuscript, as well as the design and usability of Epydemix, have been appreciated. Our goal was precisely to provide a transparent and flexible framework that supports both research and practical applications in epidemic modeling, and we are glad to see that this has been recognized.
